# “Conheça o seu Inimigo e a si Mesmo”. Risco Cardiovascular na Pesquisa Nacional de Saúde

**DOI:** 10.36660/abc.20210105

**Published:** 2021-03-03

**Affiliations:** 1 Universidade de São Paulo São PauloSP Brasil Universidade de São Paulo, São Paulo, SP – Brasil.

**Keywords:** Doenças Cardiovasculares/mortalidade, Epidemiologia, Fatores de Risco, Obesidade, Envelhecimento

A Arte da Guerra, de Sun Tzu^1^ é uma obra-prima sobre estratégia militar publicada há cerca de 2.500 anos. Em sua obra, Tzu afirma: “Se você conhece o inimigo e conhece a si mesmo, não precisa temer o resultado de cem batalhas.” Embora não sejamos generais do exército, os ensinamentos de Tzu também podem ser úteis na ciência cardiovascular. Eles reforçam a necessidade de compreender o quadro atual da epidemiologia cardiovascular em nossa sociedade e como ela evolui, como uma arma primária para determinar como os recursos e esforços podem ser aplicados de forma mais eficiente.

As doenças cardiovasculares continuam sendo uma das principais causas mundiais de morte e incapacidade. Embora as pesquisas nas últimas décadas tenham melhorado consideravelmente nosso conhecimento sobre os principais fatores da epidemiologia das doenças cardiovasculares, as altas taxas de morbimortalidade cardiovascular geram um quadro muito heterogêneo ao redor do mundo.^2^ No nosso país, por exemplo, nas últimas décadas, observamos uma queda acentuada do tabagismo em todos os estados brasileiros.^3^ Entretanto, também observamos tendências crescentes para outros fatores de risco como diabetes^4^ e outras alterações metabólicas relacionadas à obesidade,^5^ assim como o envelhecimento da população, produzindo impactos mistos nas taxas de morbimortalidade cardiovascular.

Em artigos publicados recentemente nos Arquivos Brasileiros de Cardiologia, tem-se dado visibilidade ao risco cardiovascular em populações específicas em nosso país. Silva et al.,^6^ estudaram 71 indivíduos com HIV (população de alto risco cardiovascular em comparação à população geral^7^) em Minas Gerais. Os participantes desse estudo tinham idade média de 47,2 anos e 53% eram homens. Verificou-se que mais de um quarto desses participantes tinha >20% de probabilidade de eventos cardiovasculares em 10 anos. Oliveira et al.,^8^ estudaram 11 pacientes consecutivos do sexo masculino com psoríase (um distúrbio inflamatório também associado a risco cardiovascular aumentado^9^) e 33 controles pareados por idade e observaram níveis significativamente mais elevados de colesterol total, colesterol LDL e proteína C reativa e uma tendência de aumento na frequência de diagnóstico de tabagismo e hipertensão em indivíduos com psoríase em comparação com controles.

Na edição atual dos Arquivos, Malta et al.,^10^ analisaram dados da Pesquisa Nacional de Saúde realizada em 2013, com subsequentes avaliações laboratoriais realizadas em 2014 e 2015. É importante frisar que esta é uma contribuição necessária para o campo. Em primeiro lugar, a metodologia adotada mostra o “panorama” do risco cardiovascular em nosso país, com uma amostra grande e representativa. Em segundo lugar, os autores apresentam informações detalhadas sobre as distribuições dos escores de risco de Framingham de acordo com características sociodemográficas de nosso país, usando ferramentas estatísticas adequadas para evitar os vieses de seleção. Por fim, o acréscimo da aferição da pressão arterial e medições laboratoriais para uma subamostra da Pesquisa Nacional de Saúde reduz consideravelmente o impacto do subdiagnóstico nos resultados. Dentre seus principais resultados, os autores estimam que 5,8% das mulheres e 21,6% dos homens em nosso país apresentam risco elevado (>20%) de eventos cardiovasculares em 10 anos.

Mais avanços são certamente necessários neste campo. Um ponto importante é que não está claro até que ponto o clássico escore de risco de Framingham é adequado para identificação de risco na população brasileira. É interessante notar que a Diretriz da ACC/AHA de 2013 sobre a Avaliação de Risco Cardiovascular^11^ identificou a necessidade de critérios de pontuação específicos para cada raça. Seus autores descrevem diferentes equações para brancos e afro-americanos e reconhecem as limitações de aplicar os mesmos cálculos para outros grupos étnicos. Pode ser que nossas estimativas de risco cardiovascular serão ainda mais precisas à medida que mais informações “locais” sejam disponibilizadas a partir de estudos de coorte de longo prazo no Brasil.^12^ Avanços nas pesquisas de epidemiologia cardiovascular nos permitirão conhecer o inimigo, nos conhecer e vencer mais batalhas.
