# Supplementary material for: “Conheça o seu Inimigo e a si Mesmo”. Risco Cardiovascular na Pesquisa Nacional de Saúde
Source: Arq Bras Cardiol. 2021 Mar 3;116(3):432–3. [Article in Portuguese] doi: 10.36660/abc.20210105 (PMC8159544; doi:10.36660/abc.20210105)
Supplement: Translation

# Translation: “Know the Enemy and Know Yourself”. Cardiovascular Risk in the National Health Survey

**Affiliations:** 1 São PauloSP Brazil Universidade de São Paulo, São Paulo, SP – Brazil.

**Keywords:** Cardiovascular Diseases/mortality, Epidemiology, Risk Factors, Obesity, Aging

The Art of War by Sun Tzu^1^ is a masterpiece about military strategy published around 2,500 years ago. In his work, Tzu states: “If you know the enemy and know yourself, you need not fear the result of a hundred battles.” Although we are not army generals, teachings from Tzu may be proven useful in cardiovascular science as well. They reinforce the need for understanding the current picture of cardiovascular epidemiology in our society and how it evolves, as a primary weapon to determine how resources and efforts may be more efficiently applied.

Cardiovascular diseases remain a major cause of death and disability worldwide. Although research in the past decades have substantially improved our knowledge about the main drivers of cardiovascular disease epidemiology, the high rates of cardiovascular morbimortality yields a very heterogeneous picture around the globe.^2^ In our country, for example, in the past few decades we have experienced a sharp decline in smoking in all Brazilian states.^3^ However, we also observed raising trends for other risk factors such as diabetes^4^ and other obesity-related metabolic changes,^5^ as well as population aging, producing mixed impacts on cardiovascular morbimortality rates.

In articles recently published in *Arquivos Brasileiros de Cardiologia*, visibility has been given to cardiovascular risk in special populations in our country. Silva et al.^6^ have studied 71 individuals living with HIV (a population with high cardiovascular risk compared to the general population^7^) in Minas Gerais. Participants in that study had a mean age of 47.2 years, and 53% were men. They found that more than one-fourth of those participants had >20% probability of cardiovascular events in 10 years. Oliveira et al.^8^ studied 11 consecutive male patients with psoriasis (an inflammatory disorder also associated with increased cardiovascular risk^9^) and 33 age-matched controls and found significantly higher total cholesterol, LDL-cholesterol and C-reactive protein levels and a trend towards higher frequencies of smoking and hypertension diagnosis in individuals with psoriasis compared to controls.

In the current issue of *Arquivos*, Malta et al.^10^ analyzed data from the National Health Survey conducted in 2013, with subsequent laboratory assessments conducted in 2014 and 2015. It is important to state that this is a necessary contribution to the field. Firstly, the adopted methodology shows the “big picture” of cardiovascular risk in our country, with a large and representative sample. Secondly, the authors present detailed information about Framingham risk score distributions according to sociodemographic characteristics in our country, using adequate statistical tools to account for sampling biases. Finally, the addition of blood pressure and laboratory measurements for a subsample of the National Health Survey substantially reduces the impact of underdiagnosis in the results. Among their main results, the authors estimate that 5.8% of women and 21.6% of men in our country have high (>20%) risk for cardiovascular events in 10 years.

Further advance is surely needed in this field. One important point is that the extent to which the classical Framingham risk score is appropriate for identifying risk for the Brazilian population is not clear. Interestingly, the 2013 ACC/AHA Guideline on the Assessment of Cardiovascular Risk^11^ identified the need for race-specific scoring criteria. Its authors describe different equations for White and African Americans and recognize the limitations of applying the same calculations for other ethnic groups. It may be true that our estimates of cardiovascular risk will be even more accurate as more “native” information becomes available from long-term cohort studies in Brazil.^12^ Advancing in cardiovascular epidemiology research will allow us to know the enemy, to know ourselves and to win more battles.
